# The Genetic Polymorphisms of *NPPA*:rs5065 and *NPPB*:rs198389 and Intermediate Phenotypes of Heart Failure in Polish Patients

**DOI:** 10.3390/ijms26104567

**Published:** 2025-05-10

**Authors:** Anna Gorący-Rosik, Mateusz Fic, Jakub Rosik, Klaudyna Lewandowska, Krzysztof Safranow, Andrzej Ciechanowicz, Iwona Gorący

**Affiliations:** 1 Department of Clinical and Molecular Biochemistry, Pomeranian Medical University, 70-111 Szczecin, Poland; anna.goracy.rosik@pum.edu.pl (A.G.-R.); ficmateusz91@gmail.com (M.F.); klaudyna.lewandowska@pum.edu.pl (K.L.); iwona.goracy@pum.edu.pl (I.G.); 2 Department of Physiology, Pomeranian Medical University, 70-111 Szczecin, Poland; jakub.rosik@pum.edu.pl; 3 Department of Biochemistry and Medical Chemistry, Pomeranian Medical University, 70-111 Szczecin, Poland; krzysztof.safranow@pum.edu.pl

**Keywords:** natriuretic peptides, genetic polymorphism, heart failure

## Abstract

Heart failure (HF) is a complex disease and a major cause of morbidity and mortality worldwide. Natriuretic peptides (NPs) are involved in the pathogenesis of HF, but their activity may be modified by polymorphisms in the genes encoding them. Aim: To examine the associations of *NPPA*:rs5065 and *NPPB*:rs198389 polymorphisms with the risk of HF and cardiovascular phenotypes in Polish patients with HF. The study group comprised 330 HF patients, and the control group comprised 206 healthy newborns. Genomic DNA was extracted from blood, and genotyping of both polymorphisms was performed using polymerase chain reaction–restriction fragment length polymorphism. There were no significant differences in the distributions of *NPPA* and *NPPB* genotypes between HF patients and controls. Within the HF group, there were no significant associations between the frequencies of type 2 diabetes, hypertension, left ventricular hypertrophy, or categories of left ventricular ejection fraction (LVEF) and the *NPPA* or *NPPB* variants. However, LVEF was significantly higher in *NPPA* CC homozygotes than in carriers of at least one T allele. The results of our study did not confirm an association between the *NPPA*:rs5065 or *NPPB*:rs198389 polymorphisms and predisposition to HF or HF intermediate phenotypes, except for LVEF.

## 1. Introduction

Heart failure (HF) is a complex disease and a major cause of morbidity and mortality worldwide [1]. In 2017, more than 64 million people suffered from HF globally, and its prevalence is expected to continue rising [2]. According to a universal consensus introduced in 2021, HF is defined as a clinical syndrome with symptoms and/or signs caused by a structural and/or functional cardiac abnormality, corroborated by elevated natriuretic peptide (NP) levels and/or objective evidence of pulmonary or systemic congestion [2,3]. Additionally, based on the left ventricular ejection fraction (LVEF), Bozkurt et al. [3] proposed classifying HF into three LVEF categories: HF with a reduced ejection fraction (HFrEF), defined as symptomatic HF with an LVEF of ≤40%; HF with a mildly reduced ejection fraction (HFmrEF), defined as symptomatic HF with an LVEF of 41% to 49%; and HF with a preserved ejection fraction (HFpEF), defined as symptomatic HF with an LVEF of ≥50%. The major causes of HF include coronary artery disease (CAD), arterial hypertension, heart valve disease, and cardiomyopathy [1].

The pathogenesis of HF is complex and primarily involves activation of the renin-angiotensin–aldosterone system and sympathetic nervous system, followed by activation of natriuretic peptides (NPs), which exert antagonistic effects against the increased activities of these systems [4,5,6]. Recently, Kuwahara [6] reviewed the role of atrial NP (ANP) and brain (or B-type) NP (BNP), which are physiologically abundant and secreted in the atria and ventricles, respectively. These NPs are of particular interest in the context of HF because of their cardioprotective effects and their increased concentrations in patients with HF [6].

The ANP or BNP precursors (preproANP or preproBNP, respectively) are encoded in humans by the *NPPA* and *NPPB* paralogous genes, which are located 9118 base pairs apart on chromosome 1q36 [7]. The *NPPA*:rs5065 polymorphism is a c.2238T>C transition that abolishes the regular stop codon (TGA). Analysis of the *NPPA* gene sequence predicts that this nonstop mutation results in an extension of preproANP translation by two additional arginines, leading to the production of mature ANP (alpha-carboxy-terminal peptide) consisting of 30 amino acids instead of 28 [8]. Rubattu et al. revealed that, in vitro, such synthetic 30-amino-acid-long ANP exerts deleterious biological effects, including increased generation of reactive oxygen species, endothelial damage, contraction of vascular smooth muscle cells, and platelet aggregation [8,9]. However, to the best of our knowledge, no reports to date have confirmed the presence of ANP with two additional arginines in vivo in humans. It is also worth noting that, as early as 1984, Greenberg et al. [10] identified these two arginines as a potential proteolytic site.

Few reports concerning the association of the *NPPA*:rs5065 polymorphism with the concentration of plasma ANP have been published, and their results appear to be conflicting [11,12,13,14,15,16]. Kato et al. [11] reported no association between the plasma ANP level and the *NPPA*:rs5065 polymorphism in 102 healthy normotensive Japanese subjects. An age- and sex-adjusted analysis conducted by Cannone et al. [12] in a random community-based sample of 1623 subjects also revealed no differences in plasma ANP concentrations regarding the *NPPA*:rs5065 polymorphism. However, they observed that the minor c.2238C allele was significantly associated with higher BNP levels in plasma [12]. On the other hand, Nannipieri et al. [13] found in a homogeneous cohort of normotensive patients with type 1 diabetes that plasma ANP levels in wild-type *NPPA*:rs5065 homozygous subjects (TT homozygotes) were significantly higher than those in c.2238T>C heterozygotes. By contrast, we found in Polish individuals with salt-sensitive hypertension that plasma ANP concentrations on a normal- or high-sodium diet were significantly higher in carriers of the *NPPA*:rs5065 minor allele (c.2238C variant) than in wild-type homozygotes [14]. Similarly, Vassalle et al. reported that, among patients with New York Heart Association (NYHA) class III to IV HF, carriers of the risk allele (CT + CC) had higher ANP concentrations than wild-type *NPPA*:rs5065 homozygotes (TT homozygotes) [15].

Despite no clear evidence for the association of *NPPA*:rs5065 with this intermediate phenotype, the results of a recent meta-analysis based on 12 studies involving more than 45,500 patients from different ethnic groups suggest that the c.2238T>C *NPPA* transition increases the risk of myocardial infarction, cerebrovascular events, and composite cardio-cerebral vascular events [16]. However, the results of another meta-analysis based on 13 case-control studies involving more than 7000 subjects suggested no significant association between the *NPPA*:rs5065 polymorphism and the risk of essential hypertension in various ethnic groups (Tunisia, China, Slovenia, Japan, Italy, and African Americans) [17].

The *NPPB*:rs198389 (c.-381T>C) promoter polymorphism is a functional variant that disrupts a transcriptionally active enhancer box, and the c.-381T>C transition is associated with a 1.8-fold higher level of promoter activity in vitro compared with the wild-type T allele [18,19]. The mutated c.-381C *NPPB* allele (minor allele) has been consistently associated with elevated mean levels of NT-proBNP and/or BNP in various ethnic groups [19,20,21,22,23], including studies that used genome-wide genotyping [24,25,26,27,28,29] or genome-wide sequencing [30]. On the other hand, Seidelmann et al. revealed in patients (n = 11,361 African Americans and whites) from the Atherosclerosis Risk in Communities (ARIC) study that the *NPPB*:rs198389 minor allele (c.-381C allele) was not only associated with elevated NT-proBNP levels throughout adult life but also with reduced blood pressure, a reduced risk of hypertension and cardiovascular mortality, and an increased lifespan [18]. Similarly, Cannone et al. in 971 patients with cardiovascular risk factors but without symptomatic HF from the St Vincent’s Screening to Prevent Heart Failure (STOP-HF) follow-up study, found that the c.-381C *NPPB* allele was associated with higher circulating levels of BNP and a favorable clinical phenotype and outcome, characterized by a lower risk of hypertension, new-onset left ventricular systolic dysfunction, and major adverse cardiovascular events [31].

Therefore, the aim of our study was to analyze the associations of *NPPA*:rs5065 and *NPPB*:rs198389 polymorphisms in Polish patients with HF with the risk of HF, as well as with cardiovascular phenotypes, including type 2 diabetes mellitus (T2DM), hypertension, left ventricular mass (LVM), and LVEF.

## 2. Results

The *NPPA*:rs5065 and *NPPB*:rs198389 genotype distributions in the HF group or in control newborns conformed to the expected Hardy–Weinberg equilibrium (*p* = 0.096 and *p* = 0.557 or *p* = 0.237 and *p* = 0.271, respectively).

For the *NPPA*:rs5065 (c.2238T>C) polymorphism, among the 85 women with HF, there were 57 (67.1%) TT homozygotes and 28 (32.9%) TC heterozygotes, with the frequency of the minor *NPPA*:c.2238C allele at 16.5% (28 of 170 alleles). Among the 245 men with HF, there were 182 (74.3%) TT homozygotes, 60 (24.5%) TC heterozygotes, and 3 (1.2%) CC homozygotes, with the frequency of the minor *NPPA*:c.2238C allele at 13.5% (66 of 490 alleles). There were no significant differences in the frequency distributions of *NPPA*:rs5065 alleles or genotypes between women and men with HF (*p* = 0.335 and *p* = 0.075, respectively).

For the *NPPB*:rs198389 (c.-381T>C) polymorphism, among the 85 women with HF, there were 24 (28.2%) TT homozygotes, 45 (53.0%) TC heterozygotes, and 16 (19.8%) CC homozygotes, with the frequency of the minor *NPPB*:c.-381C allele at 45.3% (77 of 170 alleles). Among the 245 men with HF, there were 88 (35.9%) TT homozygotes, 111 (45.3%) TC heterozygotes, and 46 (18.8%) CC homozygotes, with the frequency of the minor *NPPB*:c.-381C allele at 41.4% (203 of 490 alleles). There were no significant differences in the frequency distributions of *NPPB*:rs198389 alleles or genotypes between women and men with HF (*p* = 0.380 and *p* = 0.171, respectively).

There were also no significant differences in the frequency distributions of *NPPA*:rs5065 and *NPPB*:rs198389 genotypes or alleles between patients with HF and control newborns (Table 1).

Except for the frequency of smoking and LVEF values, there were no significant differences in clinical and echocardiographic variables among patients with HF regarding the *NPPA*:rs5065 (c.2238T>C) polymorphism. The prevalence of smoking in wild-type *NPPA*:rs5065 homozygotes (TT homozygotes) was significantly higher than that in patients carrying at least one mutated c.2238C allele (TC or CC genotypes). Conversely, the LVEF in patients homozygous for the mutated c.2238C *NPPA* allele was significantly higher than that in patients carrying at least one wild-type *NPPA*:rs5065 allele (TT or TC genotypes) (Table 2). However, there were no significant differences in any of the clinical or echocardiographic variables analyzed among patients with HF regarding the *NPPB*:rs198389 polymorphism (Table 3).

There were no significant differences in the frequency distributions of *NPPA*:rs5065 and *NPPB*:rs198389 genotypes or alleles between patients with HF and hypertension and those without hypertension (Table 4). In addition, no association was found between the *NPPA*:rs5065 and *NPPB*:rs198389 polymorphisms and left ventricular hypertrophy in the patients with HF (Table 5).

Finally, there were no significant differences in the frequency distributions of *NPPA*:rs5065 and *NPPB*:rs198389 genotypes or alleles among the studied patients regarding LVEF-based HF categories (Table 6).

## 3. Discussion

The results of our study showed no significant differences in the frequency distributions of either polymorphism between patients with HF and healthy newborns as the control group. In addition, within the HF group, there were no significant associations between the *NPPA*:rs5065 or *NPPB*:rs198389 genotypes or alleles and the other analyzed variables except for smoking prevalence and LVEF values in relation to *NPPA* variants. It is worth noting that the *NPPA*:rs5065 and *NPPB*:rs198389 polymorphisms were analyzed by us individually. This approach is justified because Newton-Cheh et al. [33] demonstrated in 2009 that there is no linkage disequilibrium between these polymorphisms despite their close proximity on chromosome 1 (chromosomal positions according to GRCh38.p14 Genome assembly: 1:11,846,011 for rs5065 and 1:11,859,214 for rs198389).

The present study has shown that the *NPPA*:rs5065 and *NPPB*:rs198389 polymorphisms are not associated with the risk of HF in Polish individuals. The frequencies of the minor alleles (C alleles) of *NPPA*:rs5065 and *NPPB*:rs198389 in patients with HF were 14.2% and 42.4%, respectively, and the frequencies in control neonates were 16.0% and 43.4%, respectively.

Larsen et al. [34] emphasized that healthy newborns are a representative sample for estimating the frequency of genetic variants in the general population. Notably, no deviations from Hardy–Weinberg equilibrium were observed in either our control newborns or patients with HF, suggesting no selection bias, population stratification, or genotyping errors [35] in the present study.

The validity of our results—excluding *NPPA*:rs5065 and *NPPB*:rs198389 as risk factors for HF—is further supported by an analysis of results from the NHGRI-EBI GWAS Catalog [36]. A search of the NHGRI-EBI GWAS Catalog (https://www.ebi.ac.uk/gwas/home, accessed on 11 November 2024) revealed no direct associations between these genetic variants and susceptibility to HF in genome-wide association studies. Similarly, results from a study by Geelhoed et al. [37] did not indicate a significant association between *NPPB*:rs198389 and HF in individuals from the FINRISK 1997 cohort. Furthermore, Pfister et al. [38] found no association between the c.-381T>C *NPPB* promoter polymorphism and HF risk in a prospective analysis of the European Prospective Investigation into Cancer and Nutrition (EPIC) Norfolk cohort, which included 23,192 participants. However, the authors did not exclude a potential association of the *NPPB*:rs198389 variant with specific subgroups of patients with HF, such as those with diabetes or hypertension [39].

By contrast, our study revealed no associations between the *NPPA*:rs5065 and *NPPB*:rs198389 polymorphisms and the risk for T2DM or primary hypertension in Polish patients with HF. Interestingly, in 2001, Nannipieri et al. [39] reported a significantly lower frequency of the *NPPA*:rs5065 mutated allele in Italian individuals with T2DM or hypertension compared with healthy controls in a clinical cohort of 1033 subjects. However, two years later, in 1288 participants from the Mexico City Diabetes Study, the same group found a higher frequency of the c.2238C *NPPA* allele in hypertensive patients compared with normotensive individuals but no significant differences in the distribution of *NPPA*:rs5065 genotypes or alleles between patients with T2DM and subjects with normal glucose tolerance [40].

In addition, we previously observed a higher frequency of systolic blood pressure at the ≥90th percentile in Polish newborns carrying the minor c.2238C *NPPA* allele than in wild-type homozygotes [32]. Conversely, in patients with CAD (n = 1810) from New Zealand, the minor *NPPA*:rs5065 allele (but not *NPPB*:rs198389) was associated with a lower history of hypertension [23]. Finally, a meta-analysis by Wang et al. [17] based on 7014 subjects of various ethnic origins from 13 case-control studies, including the Italians analyzed by Nannipieri et al. in 2001 [39], indicated no association between the *NPPA*:rs5065 polymorphism and the overall risk of primary hypertension.

The results of a meta-analysis based on 49,279 Europeans, including 10,239 subjects from the earlier meta-analysis by Meirhaeghe et al. clearly confirmed a decreased risk of T2DM in individuals homozygous for the minor allele (c.-381C) of the *NPPB*:rs198389 polymorphism [19]. This association between the *NPPB*:rs198389 minor variant and a reduced risk of T2DM was further supported by a meta-analysis conducted by Pfister et al. [41], which included a total of 23,382 individuals with diabetes and 57,898 controls, incorporating all subjects analyzed previously in the meta-analysis by Choquet et al. [42]. In addition, Everett et al., using a prospective case-cohort approach within the Women’s Health Study, reported a per-allele hazard ratio for T2DM of 0.92 (95% confidence interval = 0.85–0.99, *p* = 0.036) for the *NPPB*:rs198389 polymorphism in an adjusted Cox model [43]. By contrast, Meroufel et al. [44] found no association between *NPPB*:rs198389 and the risk of T2DM in an Algerian population.

Reports concerning the association of *NPPB*:rs198389 with blood pressure or hypertension are scarce [23,28,32]. As previously cited, Ellis et al. [23] found no association between the *NPPB*:rs198389 polymorphism and a lower history of hypertension in New Zealand patients with CAD. However, Musani et al. [28] reported an association between *NPPB*:rs198389 and systolic blood pressure in Black/African-American individuals as part of the COGENT consortium. In addition, we previously observed in Polish neonates that the frequency of systolic blood pressure at the ≥90th percentile was significantly higher in *NPPB*:rs198389 CC homozygotes than in those homozygous for the wild-type allele (TT homozygotes) [32].

There are also only a few reports focused on analyzing the association between *NPPA*:rs5065 and *NPPB*:rs198389 polymorphisms with LVM or left ventricular hypertrophy [32,45]. In the present study, we found no association between *NPPA*:rs5065 and LVM in patients with HF, which is consistent with the results of a study conducted in 203 hypertensive Italian individuals [45] and our previous analysis of 206 healthy Polish neonates [32]. However, we also found no association between LVM and *NPPB*:rs198389 variants, which contrasts with the results of our earlier study in newborns [32] and the findings reported by Musani et al. in White individuals from the EchoGEN consortium [28].

Finally, we found no significant associations between *NPPB*:rs198389 polymorphisms and LVEF or the LVEF categories defined by Bozkurt et al. [3]. However, the LVEF in patients homozygous for the minor *NPPA* C allele was significantly higher than in patients carrying at least one wild-type (T) allele. By contrast, Wu et al. [46], in a group of 747 consecutive Southern Han Chinese patients with CAD, found no significant differences in the frequency distributions of *NPPA*:rs5065 genotypes or alleles between individuals with a reduced LVEF (201 patients with an LVEF of ≤45%) and those with a preserved LVEF (546 patients with an LVEF of >45%).

The major limitations of our study, which reduce the ability to draw reliable conclusions, include differences in the frequencies of both polymorphisms (especially *NPPA*:rs5065) among various ethnic groups and the relatively small size of the analyzed sample. It should be noted that, of the 13 studies included in the meta-analysis by Wang et al. [17], only the analysis by Rutledge et al. [47] conducted in African Americans revealed an association of *NPPA*:rs5065 (specifically in the co-dominant model: TC versus TT) with the risk of hypertension. The frequency of the *NPPA*:rs5065 minor allele in normotensive controls, reported as 38.6% by Rutledge et al. [47], is consistent with its prevalence (41.8%) in individuals of African ancestry from the 1000 Genomes Project (https://www.ensembl.org/Homo_sapiens/Variation/Population?db=core;r=1:11845511-11846511;v=rs5065;vdb=variation;vf=377, accessed on 15 March 2025). On the other hand, in addition to the study by Rutledge et al. [47], five other studies included in the meta-analysis by Wang et al. [17] had smaller sample sizes than our current report. The simulation we performed for a significant association of the *NPPB*:rs198389 polymorphism (using the recessive inheritance model for the minor C allele) with hypertension using Open Epi (www.openepi.com), a free open-source software (version 3.01.) for statistical analysis, indicated that the required minimum sample size for 80% statistical power and a 5% type I error rate (α) would be 533 hypertensive patients with HF and 102 normotensive patients with HF (compared with 277 and 53, respectively, in our current study).

## 4. Materials and Methods

### 4.1. Patients

Both the eligibility criteria and the recruitment process of patients for our current study have been described in detail previously [1,48]. Briefly, the study group consisted of 330 patients (245 men and 85 women) with chronic HF of NYHA functional class I to IV. This group included 220 patients (170 men and 50 women) with heart failure caused by coronary heart disease, 42 patients (24 men and 18 women) with HF caused by valvular disease, and 68 patients (51 men and 17 women) with HF due to both coronary heart disease and valvular disease. The control group comprised 206 full-term healthy newborns (114 male neonates and 92 female neonates) genotyped for *NPPA*:rs5065 and *NPPB*:rs198389 genetic variants in 2015 [32]. All patients with HF underwent transthoracic echocardiography as described previously [1,48], and their clinical and laboratory data were obtained from their medical records. The study protocol was approved by the local bioethics committee at the Pomeranian University in Szczecin, and all recruited HF individuals or parents of all control newborns provided written informed consent to participate in the study. Both patients with HF and control newborns were of Polish nationality, of European descent, and lived in the Polish Western Pomerania region.

### 4.2. Genotyping

Genomic DNA was isolated from whole blood using the QIAamp Blood DNA Mini Kit (QIAGEN, Hilden, Germany). Genotyping of the *NPPA*:rs5065 (c.2238T>C) and *NPPB*:rs198389 (c.-381T>C) polymorphisms was carried out using polymerase chain reaction (PCR)-restriction fragment length polymorphism according to previously described methods [32]. Briefly, amplification of a 132-base pair (bp) *NPPA* sequence, including the rs5065 polymorphism, was performed by PCR using PCR Master Mix (2X) (Thermo Fisher Scientific, Waltham, MA, USA) with 5′-GGCACACTCATACATGAAGCTGACTTT-3′ as the forward primer and 5′-GCAGTCTGTCCCTAGGCCCA-3′ as the reverse primer. *NPPA* amplicons were subsequently digested with the ScaI restriction enzyme (Thermo Fisher Scientific), yielding restriction fragments of 78 and 54 bp for the c.2238T allele or remaining uncut for the c.2238C allele. Amplification of a 500-bp *NPPB* sequence, including the rs198389 polymorphism, was performed by PCR using PCR Master Mix (2X) (Thermo Fisher Scientific) with 5′-GCCGGGGCTGTTTTCGCTGTGAGT-3′ as the forward primer and 5′-CGGAGGCTGCTGCTGCTGCTTCTG-3′ as the reverse primer (both *NPPA* and *NPPB* primers were from TIB MOL BIOL, Poznań, Poland). *NPPB* amplicons were subsequently digested with the EcoRII restriction enzyme (Thermo Fisher Scientific), yielding restriction fragments of 238, 133, 98, and 31 bp for the c.-381T allele or 371, 98, and 31 bp for the c.-381C allele. Restriction products were electrophoretically separated in 3% agarose gels and visualized by staining with Midori Green (Nippon Genetics Europe, Düren, Germany).

### 4.3. Statistical Analyses

Parameters were compared between groups using the chi-square test or Fisher’s exact test for qualitative variables and the Kruskal–Wallis test or Mann–Whitney test for quantitative variables, as appropriate. Possible deviations of *NPPA*:rs5065 or *NPPB*:rs198389 genotype frequencies from the Hardy–Weinberg equilibrium were assessed using Fisher’s exact test. The strength of the association of qualitative variables with genotypes and alleles was described as an odds ratio with a 95% confidence interval. Two-tailed tests with *p* < 0.05 were considered statistically significant. Calculations were performed using a data analysis software system (Statistica, version 13, TIBCO software, Palo Alto, CA, USA).

## 5. Conclusions

The results of our study conducted on Polish individuals did not confirm an association between the *NPPA*:rs5065 and *NPPB*:rs198389 genetic polymorphisms and predisposition to HF or HF-intermediate phenotypes except for LVEF. These findings further highlight the impact of ethnic background on the prevalence of risk alleles and their potential association with clinical phenotypes.

## Figures and Tables

**Table 1:** Frequency distribution of *NPPA*:rs5065 and *NPPB*:rs198389 genotypes and alleles in patients with heart failure (HF) and in control newborns.

Polymorphism	HF Patients (n = 330)		Control Newborns ^a^ (n = 206)		*p* ^b^	Compared Genotypes or Alleles	OR (95% CI)	*p* ^a^
	N	(%)	n	%				
*NPPA* genotype								
TT	239	(72.4)	143	(69.4)	0.678	CC + CT vs. TT	0.86 (0.59–1.27)	0.454
TC	88	(26.7)	60	(29.1)		CC vs. CT + TT	0.62 (0.12–3.10)	0.680
CC	3	(0.9)	3	(1.5)		CC vs. TT	0.60 (0.12–3.00)	0.676
*NPPA* allele								
T	566	(85.8)	346	(84.0)	0.427	C vs. T	0.87 (0.62–1.22)	0.427
C	94	(14.2)	66	(16.0)				
*NPPB* genotype								
TT	112	(33.9)	62	(30.1)	0.445	CC + CT vs. TT	0.84 (0.58–1.22)	0.356
TC	156	(47.3)	109	(52.9)		CC vs. CT + TT	1.13 (0.71–1.78)	0.597
CC	62	(18.8)	35	(17.0)		CC vs. CT	0.98 (0.58–1.64)	0.920
*NPPB* allele								
T	380	(57.6)	233	(56.6)	0.742	C vs. T	0.96 (0.75–1.23)	0.742
C	280	(42.4)	179	(43.4)				

^a^ Results previously published in [32]. ^b^ chi-square test. Abbreviations: CI, Confidence Interval, HF, Heart Failure; *NPPA*, Natriuretic Peptide A gene; *NPPB*, Natriuretic Peptide B gene; OR, Odds Ratio.

**Table 2:** Characteristics of HF patients in regard to *NPPA*:rs5065 polymorphism.

Variable	*NPPA*:rs5065 (c.2238T>C) Genotype			*p* ^a^	*p _D_* ^b^	*p _R_* ^b^
	TT (n = 239)	TC (n = 88)	CC (n = 3)			
Age, years	66 (61:70)	67 (63:73)	68 (67:77)	0.125	0.062	0.270
BMI, kg/m^2^	29.0 (25.8:31.8)	29.0 (26.0:32.1)	29.7 (27.7:33.9)	0.766	0.767	0.526
Males, n (%)	182 (76.1)	60 (68.2)	3 (100.0)	0.204	0.199	0.306
Smokers, n (%)	87 (36.4)	19 (21.6)	1 (33.3)	0.040	0.013	0.973
T2DM, n (%)	87 (36.4)	30 (34.1)	1 (33.3)	0.925	0.693	0.930
Hypertension, n (%)	201 (84.1)	73 (82.9)	3 (100.0)	0.726	0.898	0.447
Echocardiographic parameters:						
IVST, cm	1.28 (1.10:1.40)	1.20 (1.10:1.35)	1.10 (1.05:1.90)	0.239	0.091	0.838
PWT, cm	1.12 (1.00:1.20)	1.12 (1.00:1.22)	1.20 (0.90:1.50)	0.901	0.864	0.657
LVEDD, cm	5.00 (4.70:5.50)	4.90 (4.60:5.20)	5.50 (5.20:5.50)	0.084	0.106	0.206
EF, %	50.0 (40.0:55.0)	45.0 (40.0:55.0)	60.0 (55.0:60.0)	0.112	0.797	0.037
LVM, g	241.8 (199.6:288.2) (194.2:287.5)	229.3 (179.0:281.4)	257.0 (187.7:448.7)	0.202	0.118	0.544
LVMI, g/m^2^	124.3 (98.6:148.4)	122.9 (92.6:143.5)	119.5 (93.7:235.0)	0.456	0.243	0.792

Quantitative variables are presented as median, lower quartile, and upper quartile (Q1:Q3). Qualitative data are presented as a number with corresponding percentage. ^a^ Kruskal–Wallis test for quantitative variables and chi^2^ test for qualitative variables. ^b^ Mann–Whitney test for quantitative variables and chi^2^ test for qualitative variables in dominant (*p* _D_) or recessive (*p* _R_) mode of inheritance for minor *NPPA*:rs5065 (c.2238C) allele. Abbreviations: BMI, Body Mass Index; EF, Ejection Fraction; HF, Heart Failure; IVST, Interventricular Septal (Wall) Thickness; LVEDD, left ventricular end-diastolic diameter; LVM, left ventricular mass; LVMI, left ventricular mass index; *NPPA*, Natriuretic Peptide A gene; PWT, posterior wall thickness; T2DM, type 2 diabetes mellitus.

**Table 3:** Characteristics of HF patients in regard to *NPPB*:rs198389 polymorphism.

Variable	*NPPB*:rs198389 (c.-381T>C) Genotype			*p* ^a^	*p _D_* ^b^	*p _R_* ^b^
	TT (n = 112)	TC (n = 156)	CC (n = 62)			
Age, years	66 (62:71)	65 (61:71)	69 (63:72)	0.165	0.659	0.060
BMI, kg/m^2^	28.2 (24.8:31.6)	29.7 (26.3:32.5)	28.9 (26.8:30.5)	0.112	0.058	0.857
Males, n (%)	88 (78.6)	111 (71.1)	46 (74.2)	0.392	0.198	0.993
Smokers, n (%)	32 (28.6)	57 (36.5)	18 (29.0)	0.319	0.284	0.527
T2DM, n (%)	41 (36.6)	53 (34.0)	24 (38.7)	0.785	0.818	0.591
Hypertension, n (%)	92 (82.1)	128 (82.0)	57 (91.9)	0.164	0.525	0.058
Echocardiographic parameters:						
IVST, cm	1.30 (1.10:1.40)	1.20 (1.10:1.39)	1.23 (1.11:1.40)	0.571	0.862	0.358
PWT, cm	1.11 (1.00:1.20)	1.15 (1.00:1.20)	1.12 (1.05:1.20)	0.762	0.771	0.462
LVEDD, cm	5.00 (4.70:5.50)	5.00 (4.60:5.50)	5.0 (4.70:5.50)	0.933	0.838	0.715
EF, %	45.0 (40.0:55.0)	50.0 (40.0:55.0)	45.0 (40.0:55.0)	0.806	0.515	0.886
LVM, g	239.9 (190.9:286.3)	241.8 (195.1:281.5)	236.3 (198.1:304.3)	0.746	0.808	0.444
LVMI, g/m^2^	121.4 (97.1:148.0)	122.8 (98.2:147.4)	133.5 (107.2:157.6)	0.402	0.650	0.177

Quantitative variables are presented as median, lower quartile, and upper quartile (Q1:Q3). Qualitative data are presented as a number with corresponding percentage. ^a^ Kruskal–Wallis test for quantitative variables and chi^2^ test for qualitative variables. ^b^ Mann–Whitney test for quantitative variables and chi^2^ test for qualitative variables in dominant (*p* _D_) or recessive (*p* _R_) mode of inheritance for minor *NPPA*:rs5065 (c.-381C) allele. Abbreviations: BMI, Body Mass Index; EF, Ejection Fraction; HF, Heart Failure; IVST, Interventricular Septal (Wall) Thickness; LVEDD, left ventricular end-diastolic diameter; LVM, left ventricular mass; LVMI, left ventricular mass index; *NPPB*, Natriuretic Peptide B gene; PWT, posterior wall thickness; T2DM, type 2 diabetes mellitus.

**Table 4:** An association of *NPPA*:rs5065 and *NPPB*:rs198389 polymorphisms with hypertension (HT) in patients with heart failure (HF).

Polymorphism	HF Patients with HT (n = 277)		HF Patients Without HT (n = 53)		*p* ^a^	Compared Genotypes or Alleles	OR (95% CI) for HT	*p* ^a^
	n	(%)	n	(%)				
*NPPA* genotype								
TT	201	(72.6)	38	(71.7)	0.725	CC + CT vs. TT	0.96 (0.50–1.84)	0.897
TC	73	(26.3)	15	(28.3)		CC vs. CT + TT	-	-
CC	3	(1.1)	0	(0.0)		CC vs. TT	-	-
*NPPA* allele								
T	475	(85.7)	91	(85.8)		C vs. T	1.39 (0.56–1.83)	0.977
C	79	(14.3)	15	(14.2)				
*NPPB* genotype								
TT	92	(33.2)	20	(37.7)	0.163	CC + CT vs. TT	0.22 (0.66–2.24)	0.524
TC	128	(46.2)	28	(52.8)		CC vs. CT + CT	2.49 (0.95–6.53)	0.057
CC	57	(20.6)	5	(9.5)		CC vs. TT	2.48 (0.88–6.97)	0.077
*NPPB* allele								
T	312	(56.3)	68	(64.2)		C vs. T	1.39 (0.90–2.13)	0.134
C	242	(43.7)	38	(35.8)				

^a^ Chi-square test. Abbreviations: CI, Confidence Interval; HF, Heart Failure; HT, Hypertension; *NPPA*, Natriuretic Peptide A gene; *NPPB*, Natriuretic Peptide B gene; OR, Odds Ratio.

**Table 5:** An association of *NPPA*:rs 5065 and *NPPB*:rs198389 polymorphisms with left ventricular hypertrophy (LVH) in patients with heart failure (HF).

Polymorphism	HF Patients with LVH (n = 214)		HF Patients Without LVH (n = 116)		*p* ^a^	Compared Genotypes or Alleles	OR (95% CI) for LVH	*p* ^a^
	n	(%)	n	(%)				
*NPPA* genotype								
TT	156	(72.9)	83	(71.5)	0.960	CC + CT vs. TT	0.93 (0.56–1.55)	0.973
TC	56	(26.2)	32	(27.6)		CC vs. CT + TT	1.08 (0.10–12.09)	0.947
CC	2	(0.9)	1	(0.9)		CC vs. TT	1.06 (0.09–11.91)	0.959
*NPPA* allele								
T	368	(86.0)	198	(85.3)		C vs. T	0.95 (0.60–1.50)	0.823
C	60	(14.0)	34	(14.7)				
*NPPB* genotype								
TT	71	(33.2)	41	(35.3)	0.700	CC + CT vs. TT	1.10 (0.68–1.77)	0.691
TC	100	(46.7)	56	(48.3)		CC vs. CT + CT	1.28 (0.71–2.33)	0.409
CC	43	(20.1)	19	(16.4)		CC vs. TT	1.31 (0.67–2.53)	0.428
*NPPB* allele								
T	242	(56.5)	138	(59.4)		C vs. T	1.27 (0.67–2.38)	0.461
C	186	(43.4)	94	(40.5)				

^a^ Chi-square test. Abbreviations: CI, Confidence Interval, HF, Heart Failure; LVH, Left Ventricular Hypertrophy; *NPPA*, Natriuretic Peptide A gene; *NPPB*, Natriuretic Peptide B gene; OR, Odds Ratio.

**Table 6:** An association of *NPPA*:rs5065 and *NPPB*:rs198389 polymorphisms in HF patients in regard to EF-based HF category.

Polymorphism	HFpEF (n = 167)		HFmrEF (n = 53)		HFrEF (n = 110)		*p* ^a^	*p* ^a^ HFpEF vs. Others	*p* ^a^ HFrEF vs. Others	*p* ^a^ HFpEF vs. HFrEF
	n	(%)	n	(%)	n	(%)	TT vs. TC vs. CC C+ vs. Others CC vs. Others	TT vs. TC vs. CC C+ vs. Others CC vs. Others	TT vs. TC vs. CC C+ vs. Others CC vs. Others	TT vs. TC vs. CC C+ vs. Others CC vs. Others
*NPPA* genotype										
TT	122	(73.1)	36	(67.9)	81	(73.6)	0.428	0.198	0.463	0.365
TC	42	(25.1)	17	(32.1)	29	(26.4)	0.722	0.795	0.727	0.915
CC	3	(1.8)	0	(0.0)	0	(0.0)	0.228	0.086	0.266	0.157
*NPPA* allele										
T	286	(85.6)	89	(84.0)	191	(86.8)	0.784	0.924	0.581	0.692
C	48	(14.4)	17	(16.0)	29	(13.2)				
*NPPB* genotype										
TT	52	(31.1)	21	(39.6)	39	(35.5)	0.509	0.299	0.889	0.613
TC	86	(51.5)	20	(37.7)	50	(45.4)	0.482	0.277	0.681	0.454
CC	29	(17.4)	12	(22.7)	21	(19.1)	0.689	0.503	0.921	0.715
*NPPB* allele										
T	190	(56.9)	62	(58.5)	128	(58.2)	0.935	0.717	0.914	0.763
C	144	(43.1)	44	(41.5)	92	(41.8)				

^a^ Chi-square test. Abbreviations: EF, Ejection Fraction; HF, Heart Failure; HFmrEF, HF with mildly reduced Ejection Fraction; HFpEF, HF with preserved Ejection Fraction; HfrEF, HF with reduced Ejection Fraction; *NPPA*, Natriuretic Peptide A gene; *NPPB*, Natriuretic Peptide B gene.

## Data Availability

The data are available from the corresponding author upon reasonable request.

## Abbreviations

The following abbreviations are used in this manuscript:

ANP	A-type Natriuretic Peptide (Arial Natriuretic Peptide)
ARIC	Atherosclerosis Risk in Communities
BMI	Body Mass Index
BNP	B-type Natriuretic Peptide
CAD	Coronary Artery Disease
COGENT	Clopidogrel and the Optimization of Gastrointestinal Events Trial
EF	Ejection Fraction
HF	Heart Failure
HFmrEF	Heart Failure with mildly reduced Ejection Fraction
HfpEF	Heart Failure with preserved Ejection Fraction
HFrEF	Heart Failure with reduced Ejection Fraction
IVST	Interventricular Septal (Wall) Thickness
LVEDD	Left Ventricular End-Diastolic Diameter
LVEF	Left Ventricular Ejection Fraction
LVH	Left Ventricular Hypertrophy
LVM	Left Ventricular Mass
LVMI	Left Ventricular Mass Index
NP	Natriuretic Peptide
*NPPA*	Natriuretic Peptide A gene
*NPPB*	Natriuretic Peptide B gene
NYHA	New York Heart Association
PCR	Polymerase Chain Reaction
PWT	Posterior Wall Thickness
RFLP	Restriction Fragment Length Polymorphism
STOP-HF	St Vincent’s Screening to Prevent Heart Failure
T2DM	Type 2 Diabetes Mellitus
